# Study of Cervical Mucus Cytology and Serum Inflammatory Biomarkers in Preterm Labour Compared to Term Pregnancy Cases in a Tertiary Care Center of Western Uttar Pradesh (UP), India

**DOI:** 10.7759/cureus.48884

**Published:** 2023-11-16

**Authors:** Sweta Sinha, Shikha Seth, Priyanka Rai

**Affiliations:** 1 Obstetrics and Gynaecology, Pramukhswami Medical College, Anand, IND; 2 Obstetrics and Gynaecology, Government Institute of Medical Sciences, Greater Noida, IND; 3 Obstetrics and Gynaecology, All India Institute of Medical Sciences, Deoghar, Deoghar, IND

**Keywords:** c-reactive protein (crp), neutrophils-to-lymphocytes ratio, inflammation, cervical mucus, alkaline phosphatase (alp), preterm labour

## Abstract

Purpose: The onset of labor prior to 37 weeks of gestation is preterm labor. Incidence ranges from 5% to 7% of live births in developed countries, but higher in developing countries (10-13%). Preterm birth is a major threat in perinatal health care, as well as a risk factor for neurological impairment and disability. Considering that infection is the major risk factor for preterm labor in rural areas, this study was performed to assess the cytological changes in the cervical mucus of normal-term and preterm labor cases.

Method: A hospital-based cross-sectional observational study was conducted in the Department of Obstetrics and Gynecology at a tertiary care center in western Uttar Pradesh (UP), India. The sample size calculated was 90. The neutrophil-to-lymphocyte ratio (NLR) in cervical mucus, along with serum inflammatory biomarkers such as CRP and serum alkaline phosphatase, were compared in both groups.

Result: The incidence of preterm labor increased with an increase in parity, and progression to preterm delivery is faster in the higher parity group. C-reactive protein (CRP) (p value = < 0.001) and serum alkaline phosphatases (taking 220IU/L as the cutoff value), as well as the NLR (p value < 0.001) in cervical mucus in preterm labor, are significantly higher than those in term labor cases, which can be used to predict preterm labor.

Conclusions: Higher levels of serum alkaline phosphatase (> 220 IU/L) and CRP positivity can be used as prognostic markers. Using a cutoff value of 5 for the NLR in the cervical mucus of preterm labor patients proved to be a highly accurate predictor (82.2%) for preterm labor diagnosis.

## Introduction

The World Health Organization defines preterm labor as the onset of labor prior to the completion of 37 weeks or 259 days of gestation. Incidence ranges from 5% to 7% of live births in developed countries, but is substantially higher in developing countries (10-13%) [1-2]. The overall prevalence of prematurity is 11.1% worldwide [3]. The preterm birth rate among Indian women is 18% and causes 28% of all early neonatal deaths [4]. Inflammation generated by infections is the primary cause of preterm labor, which causes higher levels of inflammatory cytokines. High concentrations of proinflammatory cytokines, such as interleukin-6 and interleukin-8 in serum, have been reported in women with symptoms of preterm labor and have been prospectively associated with preterm birth. Infection anywhere stimulates inflammatory response and many mediators, such as C-reactive protein (CRP) and alkaline phosphatase, are increased in serum [5-8]. Preterm labor is classified into the following three categories [9]:

Threatened preterm labor: Uterine contraction in the absence of cervical changes.

Early preterm labor: Uterine contraction with cervical dilatation 1-3 cm and effacement less than 80%.

Advanced preterm labor: Uterine contraction with cervical dilatation of more than 3 cm and effacement of more than 80%.

Preterm labor is the major challenge in perinatal health care as the majority of perinatal deaths occur in preterm infants, and this cohort is at high risk for neurological impairment and long-term disability long term. Numerous methods have been tried for the prediction of preterm labor, but there is as yet no definitive, non-invasive option available that is simple, feasible, and cost-effective.

This study was planned to assess the cytological changes in the cervical mucus of normal term and preterm labor cases, with the goal of developing a model for predicting preterm labor on the basis of OPD and serum inflammatory biomarkers.

## Materials and methods

A hospital-based cross-sectional observational study was conducted in the Department of Obstetrics and Gynecology at a tertiary care center in Western Uttar Pradesh (UP), India, over a study period of 18 months. Considering the prevalence of preterm labor as 6-15% in developing countries, the sample size was calculated (assuming a 5% error) by the formula 4 p q / d2, yielding a sample size of 90. Thus, 90 preterm labor and 90 term cases were enrolled in the study.

Inclusion criteria 

Pregnant females, 20-45 years old, both primigravida and multigravida cases, with singleton pregnancies and who were clinically identified with preterm labor pains were included; women of the same age and parity profile without any risk factors or complications who had attained 37 weeks of gestational age were taken as controls for the comparison of results.

Exclusion criteria

Females who had a history of preterm labor pains in the past were excluded from cases who were not sure of their last menstrual period (LMP) and had no first-trimester USG for confirmation of gestational age. Subjects with a history of premature preterm rupture of membranes (PPROM), antepartum hemorrhage, collagen disorder, and other major medical disorders (e.g., diabetes mellitus, thyroid disorder, severe anemia), who had undergone any cervical procedure, and chronic smokers were excluded. Polyhydramnios, multiple gestation, and cases with known gross congenital malformations were also excluded.

Ninety antenatal females diagnosed with preterm labor were enrolled in the study as per the above criteria. For comparison, 90 matched term labor (i.e., greater than 37 weeks) cases were also enrolled as controls. Each subject was allotted a specific enrolment number.

Gestational age was calculated from the date of the first day of the LMP; if not known, it was confirmed by the available first-trimester ultrasonography. A detailed history was taken obtaining obstetric and menstrual history, demographic and personal information, and past medical and surgical history, followed by a complete head-to-toe general examination, along with per abdomen, per speculum, per vaginal examinations. Routine antenatal investigations, along with CRP and serum alkaline phosphatase, were sent. CRP was analyzed by latex agglutination slide test (manufactured by Recombigen Laboratories, New Delhi). All the findings of the study group and control group cases were properly noted on the prepared proforma for further analysis and comparison.

Methodology

With the help of a plastic pipette, cervical mucus was aspirated prior to per vaginal examination for each study participant and taken to a glass slide to form a smear, which was air-dried and then sent for cytological examination to the Department of Pathology. Each smear of cervical mucus was stained with Leishman stain and analyzed for the presence of different inflammatory cells. The percentages of neutrophils and lymphocytes in each individual smear were quantified under a microscope and noted. The average neutrophil-to-lymphocyte ratio (NLR) was calculated for comparison in both groups.

Statistical analysis

All data collected were tabulated and statistically analyzed using appropriate statistical methods. Term and preterm case results were compared taking a p value of less than 0.05 as significant. Sensitivity, specificity, positive predictive value (PPV), and negative predictive value (NPV) of CRP positivity and raised serum alkaline phosphatases for predicting or diagnosing the preterm cases were evaluated. Cervical mucus cytological evaluation results were also analyzed in detail, giving special consideration to the presence of lymphocytes and neutrophils and their ratio in the two different groups.

## Results

Ninety antenatal females diagnosed with preterm labor were enrolled in the study as per inclusion and exclusion criteria. For comparison, 90 matched term labor (greater than 37 weeks) cases were also enrolled as controls. Each subject was allotted a specific enrolment number. Table 1 shows the distribution of cases according to age cohort and parity. Fifty percent of females with preterm labor were in the age group 25-30 years, and 58.9% of females with labor at term were in the age group 25-30 years. The mean age of females in preterm labor and labor at term was 26.53 ± 4.086 and 26.1 ± 3.061, respectively. The p value in each age group suggests that the age-wise distribution of cases in both groups was similar. The maximum number of cases in the study occurred in the 25-30 years cohort, followed by the 18-24 years cohort. In the preterm group, a plurality (26.7%) of females were para 1, while 37.8% in the term group were in the P0 category. The mean parity of females in the preterm labor group and the term group were 1.6 ± 1.3 and 1.15 ± 1.169, respectively. The maximum number of cases (n = 56, or 31.1%) belonged to the nullipara/primigravida category in our study. The p value in each row suggests the distribution of study group cases and comparison groups was similar. In the preterm group, a plurality (26.7%) of females were para 1, while 37.8% in the term group were in the P0 category. The mean parity of females in the preterm labor group and the term group were 1.6 ± 1.3 and 1.15 ± 1.169, respectively. The maximum number of cases (n = 56, or 31.1%) belonged to the nullipara/primigravida category in our study. The p value in each row suggests the distribution of study group cases and comparison group were similar.

**Table 1:** Distribution of study cases based on age cohort and parity *Fisher's exact test; #Independent-sample t test; +Pearson chi2 test

		Preterm labor	Normal labor	Total	P value
Age Category	18–24 years	32 (35.6%)	30 (33.3%)	62 (34.4%)	0.337*
	25–30 years	45 (50%)	53 (58.9%)	98 (54.4%)	
	31–35 years	11 (12.2%)	7 (7.8%)	18 (10%)	
	36–40 years	2 (2.2%)	0 (0)	2 (1.1%)	
	Mean ± SD	26.53 ± 4.09	26.10 ± 3.06		0.42#
Parity	P0	22 (24.4%)	34 (37.8%)	56 (31.1%)	0.087 +
	P1	24 (26.7%)	24 (26.7%)	24 (26.7%)	
	P2	20 (22.2%)	20 (22.2%)	40 (22.2%)	
	>=P3	24 (26.7%)	12 (13.3%)	36 (20%)	

As in Table 2, 27.8% of females with threatened preterm labor were antenatal patients who had no previous delivery, 36.6% of females with early preterm labor were those having one parity, and 38.5% of females with advanced preterm labor were those having parity more than or equal to 3. The mean parity of females with threatened, early, and advanced preterm labor was 1.6 ± 1.3, 1.51 ± 1.14, and 1.84 ± 1.77, respectively. Higher mean parity was noted in advanced preterm labor cases.

**Table 2:** Parity-based distribution of preterm labor cases

Parity	Threatened preterm Labor	Early Preterm Labor	Advanced Preterm Labor	Total
P0	10 (27.8%)	8 (19.5%)	4 (30.8)	22 (24.4%)
P1	6 (16.7%)	15 (36.6%)	3 (23.1%)	24 (26.7%)
P2	10 (27.8%)	9 (22%)	1 (7.7%)	20 (22.2%)
>=P3	10 (27.5%)	9 (22%)	5 (38.5%)	24 (26.7%))
Mean ± SD	1.64 ± 1.31	1.51 ± 1.14	1.85 ± 1.77	
Total	36	41	13	90

CRP positivity indicates the presence of agglutination, and this indicates CRP content of equal or more than 6 mg/L in undiluted serum specimens. A significant relationship in CRP positivity with preterm labor (p value ≤ 0.001) was found by applying a chi-square test for significance. Table 3 shows that 25.6% of women with preterm labor were CRP positive, compared with only 3.3% of women with labor at term. The overall sensitivity of the test was poor, only 25.5%; therefore, is not a good biomarker for predicting preterm labor in all antenatal cases, but it can be used in high-risk cases as a prognostic marker. Taking the cutoff value for serum alkaline phosphatase level as 220 IU/L, as shown in Table 3, the level was raised in 61.9% of cases of preterm labor but also in 38.1% of term labor cases; hence, it cannot be considered as a good predictive or diagnostic marker for preterm labor.

**Table 3:** Correlation (association) of inflammatory biomarkers (CRP and serum alkaline phosphatases) among the study cases Pearson's chi-square test applied CRP positivity in preterm labor: Sensitivity: 25.56%, Specificity: 96.67%, PPV: 88.46%, NPV: 56.49% Raised serum alkaline phosphatases for preterm labor: Specificity: 73%, Sensitivity: 43%, PPV: 61%, NPV: 56.4%

		Preterm labor	Normal labor	Total	P value
CRP (mg/L)	Positive	23 (25.6%)	3 (3.3%)	26 (14.4%)	< 0.001
	Negative	67 (74.4%)	87 (96.7%)	154 (85.6%)	
Serum Alkaline Phosphatase (IU/L)	≤ 220	51 (56.7%)	66 (73.3%)	117 (65%)	0.019
	> 220	39 (43.3%)	24 (26.7%)	63 (35%)	
	Mean ± SD	223.97 ± 26.07	215 ± 20.71	NS	

As depicted in Table 4, 11.1% of women with threatened preterm labor were CRP positive, as were 31.7% of women with early preterm labor and 46.2% of women with advanced preterm labor. Applying the chi-square test for significance, there was a significant difference in the positivity of CRP in these three groups (p value = 0.022), which indicates that positive CRP is suggestive of early or advanced stages of preterm labor, and thus it can be helpful in explaining prognosis.

**Table 4:** Distribution of CRP positivity in the preterm labor cases

CRP (mg/L)	Threatened Preterm Labor	Early Preterm Labor	Advanced Preterm Labor	Total
Positive	4 (11.1%)	13 (31.7%)	6 (46.2%)	23 (25.6%)
Negative	32 (88.9%)	28 (68.3%)	7 (53.8%)	67 (74.4%)
Total	36	41	13	90

Samples of mucus collected were smeared on glass slides, air-dried, and assessed for cellular components after Leishman staining. Table 5 shows the mean ± SD of lymphocytes and neutrophils in preterm labor and term labor cases, suggesting that neutrophils were significantly higher in preterm cases, while lymphocytes predominated in term cases. The difference in the mean ± SD in the preterm and term labor groups was significant; the p value was < 0.001, as depicted in Table 5, suggesting that the NLR in cervical mucus is significantly high in preterm cases and can be used for the prediction of preterm labor. (N is the number of cases.)

**Table 5:** Cervical mucus cellular components in study groups and distribution of cases according to the neutrophil-to-lymphocyte ratio Independent sample t-test

Groups		N	Mean	SD	P value
Lymphocytes	Preterm labor	90	18.6667	8.1004	<0.001
	Normal labor	90	26.772	7.35	
Neutrophils	Preterm labor	90	81.111	8.1343	<0.001
	Normal labor	90	73.2778	7.35	
NLR	Preterm labor	90	5.64	3.68	<0.001
	Normal labor	90	3.19	1.75	

The distribution of the NLR in cervical mucus taking cutoff as 4 and 5 in study cases is shown in Table 6, which was clinically significant as suggested by the p value. Considering the NLR of 5 as a cutoff value, diagnostic efficacy was highly specific (82.2%), but the sensitivity was only 51.1%. In another calculation taking the cutoff of the N/L ratio as 4, the sensitivity increased to (68.8%) while the specificity remained fairly high at (74.4%). Thus, as per our results, a cutoff value of cervical mucus N/L ratio of 4 can be utilized effectively in assessing the risk of preterm labor.

**Table 6:** Neutrophil-to-lymphocyte ratio in cervical mucus in the study cases Taking the cutoff value of the NLR as 5: Sensitivity: -51.1%, Specificity: -82.2%, PPV: -74.19%, NPV: -62.71% Taking the cutoff value of the NLR as 4: Sensitivity: -68.8%, Specificity: -74.4%, PPV: -72.94%, NPV: -70.52%

NLR in cervical mucus		Preterm labor	Normal labor	P value
Taking cut off as 5	<5	44 (48.8%)	74 (82.22%)	<0.007
	>=5	46 (51.1%)	16 (17.77%)	
Taking cut off as 4	<4	28 (31.11%)	67 (74.44%)	<0.001
	>=4	62 (68.88%)	23 (25.55%)	

## Discussion

Participants were divided into two groups: 90 women with preterm labor as study cases and 90 women at term with normal labor as controls. The mean age of the participants in this study was 26.32 ± 3.61 years, almost comparable between the case and control groups (p value = 0.307). The largest group of women in both the case and control groups were aged 25-30 years, with the second-largest group being 18-24 years, this span representing the peak reproductive age. This was comparable to Health and Family Welfare Statistics 2019-2020 (age-specific fertility rates) [10], which mentioned that age-specific fertility rated the highest in the age group 25-29 years, followed by the age group 20-24 years. The mean parity in the case and control groups was 1.61 ± 1.30 and 1.16 ± 1.17, respectively, suggesting no significant difference in the distribution of participants according to parity across both groups (p value = 0.199). A majority of women in both the case and control groups belonged to the primigravida or para-one groups.

As noted in Table 2, the mean parity of females with threatened, early, and advanced preterm labor groups were 1.6 ± 1.3, 1.51 ± 1.14, and 1.84 ± 1.77, respectively. Higher mean parity was noted in advanced preterm labor cases, suggesting that labor progresses a bit faster in high-parity cases. This result corresponds with the findings of a 2017 retrospective cross-sectional study by Halimi et al., in which 810 cases with a mean age of 28.33 ± 6.1 years were evaluated. Multivariate logistic regression analysis was used to assess multiparity as a risk factor for preterm labor; the odds ratio was 21.8% (95% CI 4.8-97.9) and the p value < 0.001 [11].

The sensitivity of CRP positivity for diagnosis of preterm labor was calculated as 25.56% and specificity as 96.7%. PPV is 88.46%, and NPV was 56.49%. This suggests a highly specific test for predicting preterm labor as a prognostic outcome, so it can be used as a marker for the onset of preterm labor in women who complain of pain during pregnancy. It was concluded that the progression of labor will definitely take place too quickly in cases with high CRP values. A case-control study involving 117 subjects delivering preterm and 117 control cases delivering at term was conducted by Pitiphat et al. [12] in which CRP was performed on both groups, and CRP levels exceeding a threshold defined in literature were associated with increased risk of preterm delivery (odds ratio = 2.55, 95% CI 1.05-6.02 for CRP less than 8 mg/L). In 2013, Halder et al. [13] conducted a prospective cohort study on a group of 280 pregnant women. CRP positivity was analyzed in the study, with 31.2% of participants showing CRP positive and 68.8% CRP negative. CRP positivity showed a positive association with preterm labor with an odds ratio of 2.384 (95% CI 1.153-4.928) and (p value = 0.01). Hvilsom et al. [14] also reported a significant association of elevated serum CRP levels with a nearly doubled increased risk of delivery before 37 weeks of gestation.

Serum alkaline phosphatase, an inflammatory biomarker, was tested in both groups. The cutoff was set at 220 U/L. Raised levels were found in 61.9% of preterm cases and only in 38.1% of controls; hence, elevated alkaline phosphatase can be used as a good marker for predicting preterm labor, with a sensitivity of 43% and a specificity of 73%. PPV is 61%, and NPV is 56.41%. Huras et al. [15] conducted a case-control study involving 83 women and concluded that the levels of alkaline phosphatase in preterm cases were higher (from 139 U/L to 368 U/L) than that in the control group (from 218 to 321 U/L), similar to our study. An increase in the level of alkaline phosphatase in serum cannot be an absolute sole marker of the risk of preterm delivery but can be used in conjunction with a significantly elevated CRP level and other specific tests.

Cervical mucus collected from preterm and term labor cases at the time of admission were analyzed for cellular changes. The mean ± SD of neutrophils in preterm labor and term labor were 81.11 ± 8.13 and 73.2778 ± 7.35, respectively (p value < 0.001). Neutrophils significantly outnumber lymphocytes in preterm labor cases as compared to term cases. The NLR in cervical mucus was significantly higher in the preterm labor group than in the control (5.67 ± 3.68 vs. 3.19 ± 1.75) with a p value < 0.001. The NLR was used as a marker for inflammatory response, which is represented by neutrophil count, host immunity, and lymphocyte count. Melissa et al. [16] found a significantly higher NLR in preterm cases than in term cases (5.9 ± 5.1 vs. 4.7 ± 3.2). Bozoklu Akkar et al. [17] conducted a prospective study to determine the relationship between the NLR and early spontaneous preterm birth and reported a significant rise in the NLR in women with spontaneous preterm birth, as compared to those with term pregnancy. Similar results were found in previous studies. Kim et al. [18] noted that NLR may be used as a predictor of placental inflammatory response (PIR), which in turn is associated with preterm labor. In 2017, Tamer et al. [19] conducted a case-control study comparing the NLR in preterm and term groups; the results suggested that an increased NLR is a marker of systemic inflammatory disease in the preterm and preeclamptic patient group (p < 0.005).

A qualitative assessment of CRP was done in this study; however, calculating the quantitative value of CRP with a cutoff value can correlate better and help in predicting the prognosis of preterm labor. This was a single-centric study, so more detailed and multicentric studies are required for better and more reliable results.

## Conclusions

In this study, we finally concluded that the peak reproductive age is 25-30 years, followed by 18-24 years. Higher mean parity cases were diagnosed mostly in advanced preterm labor because labor progress a bit faster in high-parity cases. Positive CRP is more suggestive of an early or advanced stage of preterm labor (p value < 0.022) and thus helpful in explaining the prognosis of cases (patients with preterm labor). Considering the cutoff value for serum alkaline phosphatase at 220 U/L, higher levels of serum alkaline phosphatase were observed in the advanced preterm labor group. CRP and raised serum alkaline phosphatase can be used as prognostic markers, as most patients with CRP positivity and raised serum alkaline phosphatase were in advanced preterm labor. Further quantitative CRP measurements could potentially identify cutoff values to render these markers more effective in analyzing and explaining the prognosis.

The NLR of 5 was taken as a cutoff value in cervical mucus, which proved to be a highly specific test (82.2%) for preterm labor diagnosis. Cytological cervical mucus testing is a simple, minimally invasive, outpatient-based test that can be done in the second and third trimesters to identify women who are at risk of preterm labor or who are in the early phase of preterm labor where it can be stopped with certain medications (tocolytics). A more detailed and larger study is required to confirm and narrow down these conclusions, but a combination of cervical mucus cytology and testing CRP and serum alkaline phosphatases can be a good, cost-effective tool for decreasing preterm labor-related complications and mortality among the poor population of developing countries.

## References

[1] Vogel JP, Chawanpaiboon S, Moller AB, Watananirun K, Bonet M, Lumbiganon P (2018). The global epidemiology of preterm birth. Best Pract Res Clin Obstet Gynaecol.

[2] Beck S, Wojdyla D, Say L (2010). The worldwide incidence of preterm birth: a systematic review of maternal mortality and morbidity. Bull World Health Organ.

[3] Walani SR (2020). Global burden of preterm birth. Int J Gynaecol Obstet.

[4] Jana A (2023). Correlates of low birth weight and preterm birth in India. PLoS One.

[5] Goldenberg RL, Hauth JC, Andrews WW (2000). Intrauterine infection and preterm delivery. N Engl J Med.

[6] Daunter B, Councilman C (1980). Cervical mucus: its structure and possible biological functions. Eur J Obstet Gynaecol Reprod Biol.

[7] Mazor M, Kassis A, Horowitz S, Wiznitzer A, Kuperman O, Meril C, Glezerman M (1993). Relationship between C-reactive protein levels and intraamniotic infection in women with preterm labour. J Reprod Med.

[8] Yoon BH, Jun JK, Park KH (1996). Serum C-reactive protein, white blood cell counts in women with preterm premature rupture of membranes. Obstet Gynaecol.

[9] Lumley J (2003). Defining the problem: the epidemiology of preterm birth. BJOG.

[10] (2023). Age specific fertility rates (ASFR) and age specific marital fertility rates (Asmfr)- SRS during 2006-2017. http://data.gov.in/resource/age-specific-fertility-rates-asfr-and-age-specific-marital-fertility-rates-asmfr-srs.

[11] Halimi AA, Safari S, Parvareshi HM (2017). Epidemiology and related risk factors of preterm labour as an obstetrics emergency. J Emerg.

[12] Pitiphat W, Gillman MW, Joshipura KJ, Williams PL, Douglass CW, Rich-Edwards JW (2005). Plasma C-reactive protein in early pregnancy and preterm delivery. Am J Epidemiol.

[13] Halder A, Agarwal R, Sharma S, Agarwal S (2013). Predictive significance of C reactive protein in spontaneous preterm delivery: a prospective cohort study. Int Reprod contracept Obstet Gynecol.

[14] Hvilsom GB, Thorsen P, Jeune B, Bakketeig LS (2002). C-reactive protein: a serological marker for preterm delivery?. Acta Obstet Gynecol Scand.

[15] Huras H, Ossowski P, Jach R, Reron A (2011). Usefulness of marking alkaline phosphatase and C-reactive protein in monitoring the risk of preterm delivery. Med Sci Monit.

[16] Melissa CL, Aaron HG, Jonathan HBS, Angel GR, James A (2018). Neutrophil to lymphocyte ratio and red blood cell distribution width levels in preterm vs term birth. J Mol Genet Med.

[17] Bozoklu Akkar O, Sancakdar E, Karakus S (2016). Evaluation of maternal serum 25-hydroxyvitamin D, paraoxonase 1 levels, and neutrophil-to-lymphocyte ratio in spontaneous preterm birth. Med Sci Monit.

[18] Kim MA, Lee YS, Seo K (2014). Assessment of predictive markers for placental inflammatory response in preterm births. PLoS One.

[19] Talmer LH, Aykanat Y, Sager FG, Olmuşçelik O, Özdemir S (2017). Status of neutrophil lymphocyte ratio and 25 hydroxyvitamin D in preeclampsia and preterm birth. Perinatal Journal.

